# Fruit and Vegetable Processing Waste as Potential Raw Material for Food Enrichment With Dietary Fiber

**DOI:** 10.1002/fsn3.70766

**Published:** 2025-08-04

**Authors:** Agnieszka Nawirska‐Olszańska, Maciej Oziembłowski

**Affiliations:** ^1^ Department of Fruit, Vegetable and Plant Nutraceutical Technology Wroclaw University of Environmental and Life Sciences Wrocław Poland; ^2^ Department of Functional Food Products Development Wroclaw University of Environmental and Life Sciences Wrocław Poland

**Keywords:** ADF, cellulose, fruit and vegetable pomace, hemicellulose, lignin, NDF, SDF

## Abstract

For many years, dietary fiber (DF) has been an underestimated component of plant foods. Currently, there is a growing appreciation of its health‐promoting properties, especially in relation to its effect on the digestive tract. For this reason, both raw materials and products with the highest possible DF content are sought. Fruit and vegetable pomace seems to be both a raw material and a waste product with these characteristics. The aim of this study was to compare the content of neutral detergent fiber (NDF) and acid detergent fiber (ADF) in pomace from the processing of apples, chokeberries, blackcurrants, raspberries, red cabbage, two strawberry varieties, and two carrot varieties. In the study, the content of NDF, ADF, and cellulose was determined using the van Soest method, and the content of soluble dietary fiber fraction SDF, hemicellulose, and lignin was calculated. The highest content of both DF fractions was shown in chokeberry pomace (NDF—90.32 g/100 g DM, ADF—54.44 g/100 g DM), and the lowest—in Flakkese carrot pomace. High content of the soluble fraction was characteristic for apple pomace and both carrot varieties. Based on the PCA analysis, the similarity of individual raw materials was found in terms of the analyzed discriminants. The features of two raw materials, i.e., blackcurrant and chokeberry, were significantly different from the others, which may have practical significance when selecting specific raw materials for processing. The PCA analysis also showed the mutual dependencies between the analyzed discriminants. Chokeberry pomace, blackcurrants, and two strawberry varieties proved to be the most suitable for the production of functional food rich in fiber due to the content of all DF fractions.

## Graphical abstract

Currently, there is a growing interest in dietary fiber (DF) due to its health‐promoting properties, especially in relation to the impact on the gastrointestinal tract. For this reason, both raw materials and products with the highest DF content are sought. Fruit and vegetable pomace seem to be both a waste raw material with such characteristics. The aim of this study was to compare the content of neutral detergent fiber (NDF) and acid detergent fiber (ADF) in pomace from the processing of apples, chokeberries, black currants, raspberries, red cabbage, two strawberry varieties, and two carrot varieties.

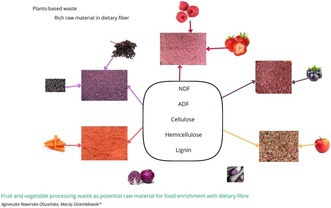

## 1 Introduction

Dietary fiber was considered for many years a ballast substance as an indigestible food ingredient. For the last several decades, however, it has been regarded as a very desirable diet component. Dietary fiber is most often defined as plant polysaccharides and lignins, resistant to hydrolysis by digestive enzymes of the human gastrointestinal tract (AACC 2001). Dietary fiber is composed of structural plant components, such as cellulose, hemicellulose, lignins, and pectins, as well as resins and waxes (Prosky 1999). Additionally, the definition has been revised to include resistant starch, although this type of starch is not among the structural components of plant cells. Similarly to other fiber components, it is not digested by humans.

Since the mid‐sixties, research has been mainly focused on physiological fiber properties in the digestive tract. Numerous studies demonstrate that DF components bind a number of substances, e.g., cholesterol and gastric juices (Jiménez‐Escrig and Sánchez‐Muniz 2000; Mittal et al. 2007). It has been proven that DF, due to its properties, plays an important role in both the prevention and treatment of diabetes, heart diseases, atherosclerosis, obesity, and both colon and colorectal cancers (Davidson and McDonald 1998; Schneeman 1998; Ferguson and Harris 2003; Slavin 2005; Ferguson 2005; Alpert 2013; Li et al. 2020; Zheng et al. 2024). It also contributes to increasing the body's immunity (Venter et al. 2022).

Epidemiological research allowed finding the relationship between low DF intake and the incidence of lifestyle diseases. For people living in highly developed countries, a daily diet low in dietary fiber is believed to be not only the cause of lifestyle diseases (atherosclerosis, obesity, diabetes, dental caries) but also of the non‐infectious gastrointestinal disorders (chronic constipation, cecitis, polyps, tumors) (Jiménez‐Escrig and Sánchez‐Muniz 2000; Sangnark and Noomhorm 2003; Mai et al. 2003; Corréa Lima and Gomes‐Da‐Silva 2005; Rodrígez et al. 2006; Alpert 2013).

Besides, pomace was demonstrated as well able to bind heavy metals, with the amount of metals bound depending on its fraction; hemicellulose and pectins had a remarkable ability to do so. Cellulose and lignins demonstrate similar properties, yet to a lesser extent, depending on where the fraction is derived from (Nawirska 2005; Borycka 2012).

Varied chemical structure of individual DF fractions determines their different activity. Water‐soluble dietary fiber (pectins, gums, certain types of hemicellulose) undergoes bacterial fermentation in the gastrointestinal tract and affects carbohydrate and lipid metabolism. Insoluble fiber (cellulose, lignins, hemicellulose) stimulates peristalsis, the same fighting constipation. It limits the growth of many types of colon cancers by promoting the growth of intestinal microflora and suppressing putrefactive bacteria (Bingham et al. 2003). Besides, insoluble DF fractions might play a role of free radical scavengers as well as deactivators of certain toxic substances in the intestinal tract. Also, they stimulate chewing and salivation, inhibiting the growth of tooth decay bacteria. By helping lower total blood cholesterol levels, mainly its LDL fraction, soluble DF fights atherosclerosis. As it prevents reabsorption of bile acids from the intestines, dietary fiber lowers the gallstone formation (Jiménez‐Escrig and Sánchez‐Muniz 2000).

Including fiber‐rich foods in your daily diet benefits your heart health and digestive health. How much dietary fiber is enough? Guidelines recommend that if you are aged 50 years or younger, men get 38 g and women 25 g daily. If you are 51 years or older, men should get 30 g and women 21 g daily according to the recommendations of the Institute of Medicine, USA National Academy of Sciences. Therefore, research is carried out into the dietary fiber content in foods (Nile and Park 2014; Sumczynski et al. 2015).

An important role of DF in health promotion and balanced nutrition makes it purposeful to enrich the diet with this ingredient. Increasing consumption of high‐fiber foods is accompanied by an increasing demand for fiber‐enriched products as well as preparations and parapharmaceuticals being its concentrated source. Such products may contain either all of the DF fractions specific to a certain plant or the isolated, individual fiber fractions: pure cellulose, plant gum, or pectin preparations.

Mostly cereal, fruit, and vegetable parts, rich in non‐digestible carbohydrates, are used for high‐fiber supplement production. Dietary fiber properties and its utility value depend on the source of origin and percentage share of individual fractions. Interactions between individual fiber components and between the fiber and other substances are equally important. High‐fiber supplements may be received from fruit and vegetable processing waste with high‐fiber content (Nawirska and Kwaśniewska 2005). Such waste utilization is convenient and cost‐effective due to the waste availability and low cost, while also important because of hard‐to‐dispose‐of waste management.

There are many works on the influence of dietary fiber on health or its content in fruits, vegetables, and cereals. (Jiménez‐Escrig and Sánchez‐Muniz 2000; Davidson and McDonald 1998; Schneeman 1998; Ferguson and Harris 2003; Alpert 2013). Nowadays, there are more and more studies on adding pomace to food to increase the content of dietary fiber. (Borderías et al. 2005; Balli et al. 2021; Elham et al. 2023; Baskaya‐Sezer 2024).

Yet, not much research has been focused so far on fruit and vegetable pomace which, as a waste product, is a dietary fiber cost‐effective and valuable source. The objective of our study was to compare the content of neutral detergent (NDF), acidic detergent (ADF) and soluble (SDF) fractions of DF as well as cellulose, hemicellulose, and lignins in pomace from the selected fruits and vegetables. These results may aid the choice of a suitable pomace type for specific high‐fiber composite preparation or their use as food additives.

## 2 Materials and Methods

### 2.1 Samples

The study material included pomace from Lobo apples, blackcurrants, elderberries, chokeberries, Polana raspberries, Alba and Elkat strawberries, as well as orange carrots Flakkese, black carrots Deep Purple, and red cabbage Langendijker. The pomace was supplied from raw material processing at the Department of Fruit, Vegetable and Plant Nutraceuticals Technology, Wrocław University of Environmental and Life Sciences.

### 2.2 Analysis

The investigations were carried out at the laboratory of the Department of Fruit, Vegetable and Plant Nutraceuticals Technology, Wrocław University of Environmental and Life Sciences. Immediately upon arrival at our laboratory, the wet material was frozen. Prior to physical and chemical determinations, it was defrosted, dried, and ground. The Van Soest procedure was used for both fractions determination using an acidic and neutral detergent. The method consists of selective separation of fiber fractions under certain conditions by treatment with surfactants (van Soest et al. 1991).

Cellulose was determined via etching with a mixture of nitric, acetic, and trichloroacetic acid. Hemicellulose content was calculated as a difference between NDF and ADF, while lignin content—a difference between ADF and cellulose. An estimated content of soluble fraction (SDF) was calculated from the equation.

(1) SDF=100−NDF

### 2.3 Statistical Analysis

The statistical software package Statistica 13.3 (StatSoft Poland) was used to analyze the received data. The data were recorded as the means ±SD and analyzed using Excel 2007. Analysis of variance was performed by ANOVA procedures. Significant differences (*p* ≤ 0.05) between the mean values were determined by Duncan's Multiple Range Test.

A PCA analysis and a correlation between the selected properties of the samples were also performed.

## 3 Discussion of Results

A lot of current research addresses DF content in raw materials. However, not many publications in the world literature have been devoted to DF content in fruit and vegetable pomace. As a waste product, it may become the cost‐effective and valuable raw material for high‐fiber supplement production (Baskaya‐Sezer 2024).

It is evident from the data presented in Table 1 that pomace is characterized by high DF content. NDF content in the fruit pomace ranged between 21.70 g/100 g DM and 90.32 g/100 g DM, while in the vegetable processing wastes it was between 15.15 g/100 g DM and 31.51 g/100 g DM. The NDF content differences were statistically significant. ADF content in the fruit pomace was also very high, namely 15.82÷54.44 g/100 g DM, and slightly lower in the pomace from vegetables, i.e., between 10.59 g/100 g DM and 31.51 g/100 g DM. Statistical analysis allowed the identification of 7 homogenous groups. The processing wastes from chokeberries, raspberries, red cabbage, and both carrot varieties formed separate homogenous groups. In contrast, pomace from blackcurrants and both strawberry varieties belonged to the same group. Fruits showed a higher content of both DF fractions, while the lowest value was determined for the apple pomace.

**TABLE 1:** Proportion of NDF, ADF, and SDF in pomace (g/100 g DM)^a^.

Pomace	NDF	ADF	SDF
Lobo apple	21.70 ± 0.07e	15.82 ± 0.12f	78.3 ± 0.53b
Blackcurrant	69.81 ± 0.61b	38.48 ± 0.24b	30.19 ± 0.22f
Black carrot Deep Purple	29.96 ± 0.17d	20.12 ± 0.20e	70.04 ± 0.57c
Carrot Flakkese	15.15 ± 0.09f	10.59 ± 0.05g	84.85 ± 0.15a
Chokeberry	90.32 ± 0.80a	54.44 ± 0.52a	9.68 ± 0.30 g
Elderberry	68.19 ± 0.59b	37.96 ± 0.28b	31.81 ± 0.31f
Polana raspberry	53.28 ± 0.48c	32.15 ± 0.24c	46.72 ± 0.38e
Red cabbage Langendijker	31.51 ± 0.27d	27.78 ± 0.18d	68.49 ± 0.47d
Alba strawberry	52.41 ± 0.38c	38.15 ± 0.29b	47.59 ± 0.38e
Elkat strawberry	54.37 ± 0.47c	37.92 ± 0.24b	45.63 ± 0.37e

^a^ Mean value of three replications; averages in columns followed by different letters indicate significant differences (*α* ≤ 0.05).

The apple pomace displayed a varied content of soluble fraction (Table 1). The largest SDF amount was recorded for the processing wastes from carrots Flakkese and apples (84.85 and 78.30 g/100 g DM, respectively). The lowest SDF content was determined for chokeberries, being only 9.68 g/100 g DM. This result significantly differs from the values received for other kinds of pomace studied. The values received by Figuerola et al. (2005) were comparable for different apple varieties; SDF varied from 56.5 to 81.6 g/100 g DM. The plum pomace SDF determined by Milala et al. (2013) ranged from 32.2% to 39.3% DM, depending on the variety. These values are only slightly higher than those now received for blackcurrants but yet slightly lower than the results for raspberries and both strawberry varieties. It is reasonable to assume carrot and apple processing wastes contain large pectin amounts, not analyzed in the current study. Additionally, the results indicate that processing wastes from chokeberries contain minor pectin amounts.

Out of all the analyzed fractions, chokeberry pomace was found to be the richest in dietary fiber. It contained the largest amounts of NDF (90.32 g/100 g DM) and ADF (54.44 g/100 g DM). Also, it was demonstrated to have the highest cellulose (39.61 g/100 g DM) and hemicellulose (35.88 g/100 g DM) content, as illustrated in Table 2. Similar results were received by Nawirska and Kwaśniewska (2005), though with different analysis methods for individual fractions. The cellulose content determined was 34.56 g/100 g DM while that of hemicellulose—30.24 g/100 g DM. In contrast, Borycka (2012) received widely varying results. The NDF content of 37.28% was reported for chokeberry processing wastes, with the cellulose of 17.73%, the amount being 2.5 times lower than in the current study. Yet, an even bigger difference was demonstrated for hemicellulose—the amount currently reported being 4 times the amount from the previous studies. However, only a minor difference was noted in the lignin content, namely 11.03% in Borycka's studies (2012) compared to the current value of 14.83 g/100 g DM.

**TABLE 2:** Proportion of cellulose, hemicellulose, and lignin in pomace (g/100 g DM)^a^.

Pomace	Cellulose	Hemicellulose	Lignin
Lobo apple	12.23 ± 0.15e	5.88 ± 0.45e	3.58 ± 0.09f
Blackcurrant	18.91 ± 0.08c	31.33 ± 1.31a	19.57 ± 0.21a
Black carrot Deep Purple	16.15 ± 0.01d	9.84 ± 0.12d	3.97 ± 0.14f
Carrot Flakkese	8.29 ± 0.01e	4.56 ± 0.22e,f	2.30 ± 0.09 g
Chokeberry	39.61 ± 0.22a	35.88 ± 0.82a	14.83 ± 0.98b
Elderberry	25.13 ± 0.44b	19.99 ± 0.11b	6.02 ± 0.54e
Polana raspberry	26.49 ± 0.71b	21.13 ± 0.98b	5.66 ± 0.18e
Red cabbage Langendijker	15.98 ± 0.89d	3.78 ± 0.27f	11.80 ± 0.54d
Alba strawberry	25.42 ± 1.02b	14.28 ± 1.07c	12.73 ± 0.62c
Elkat strawberry	24.96 ± 0.78b	16.45 ± 0.21c	12.96 ± 0.55c

^a^ Mean value of three replications; averages in columns followed by different letters indicate significant differences (*α* ≤ 0.05).

Additionally, processing wastes from blackcurrants were similarly rich in DF and contained: NDF—69.81 g/100 g DM, ADF—38.48 g/100 g DM, cellulose—18.91 g/100 g DM, hemicellulose—31.33 g/100 g DM and lignin—19.57 g/100 g DM (the highest received for any lignin fraction in the study).

These results are partially in line with the values received by Nawirska and Kwaśniewska (2005): cellulose and hemicellulose content was comparable (12.0 and 25.3 g/100 g DM, respectively), however the lignin content was over 3 times higher (59.3 g/100 g DM). Subtle differences in the individual fraction content were reported for the pomace from both strawberry varieties. Though bigger differences were demonstrated for the hemicellulose content, they had no statistical significance.

The apple pomace demonstrated the lowest DF content among the analyzed pomace samples. The NDF value received was two times lower than that obtained by previous unpublished research, namely 43.22% DM. This discrepancy could be related to the variety studied, as illustrated by Figuerola et al. (2005), whose results confirm differences in TDF content in the pomace depending on the apple variety (from 89.8 to 60.7 g/100 g DM). Cellulose, hemicellulose, and lignin content were not in line with the previous results by Nawirska and Kwaśniewska (2005). The amount of cellulose was three times higher, of hemicellulose—four times higher, and that of lignin—five times higher than the current values. However, Nawirska and Kwaśniewska (2005) used the industrial processing wastes from the Fruit Processing Plant of Prusice (Poland) for the analysis, which came from the unselected varieties.

Vegetable pomace showed slightly lower content of individual DF fractions than the majority of fruit processing wastes studied. Comparable values were obtained for the pomace from red cabbage and carrot Deep Purple. NDF and cellulose content constituted a homogenous group, while the remaining fractions formed separate homogenous groups. In the literature, reports on DF content in red cabbage pomace or in a fresh vegetables could not be found. Known is, however, the DF content in the white cabbage amounting to 14.8 g/kg of fresh matter and ADF accounting for 11.6 g/kg of fresh matter (Rahman et al. 2002).

Out of all the processing wastes analyzed, the smallest amounts of DF were detected in the two carrot varieties and apples. They were characterized by small amounts of lignins (2.30 and 3.97 g/100 g DM for carrot varieties and 3.58 g/100 g DM for apples). These results significantly differ from the study by Nawirska and Kwaśniewska (2005), where the lignin content in the carrot pomace was determined as 32.2 g/100 g DM. The reason behind that difference, similarly to the case of apple pomace, may be the unselected industrial raw material as well as the processing method. The carrot pomace used for studies in 2005 came from the Fruit and Vegetables Processing Plant Agros‐Fortuna, Łowicz (Poland). Such major discrepancies may result not only from the determination method applied but also from raw material varieties as well as the pressing process itself. The chemical composition is known from the literature to depend on the variety; thus, DF content must be variety‐dependent as well. In the studies by Rahman et al. (2002) the NDF and ADF content in fresh carrot amounted to 13.5 and 11.6 g/kg of fresh matter, respectively.

At generally low DF content in both carrot varieties, the individual fraction content in the pomace from black carrot was significantly higher than in the carrot Flakkese. Black carrot as well as its pomace is rich in anthocyanins. Thus, despite relatively low DF content, it should become a valuable source for functional food ingredients.

Based on the results obtained, a PCA analysis was also performed. The input data referred to 10 cases (pomace) and 6 variables as average values from Tables 1 and 2. PCA analysis showed that the first two PCA factors (i.e., PCA 1 and PCA 2) explain 86.29% of the total variance. The eigenvalues were 5.177 and 0.548 for PCA 1, PCA2, respectively.

**FIGURE 1:**
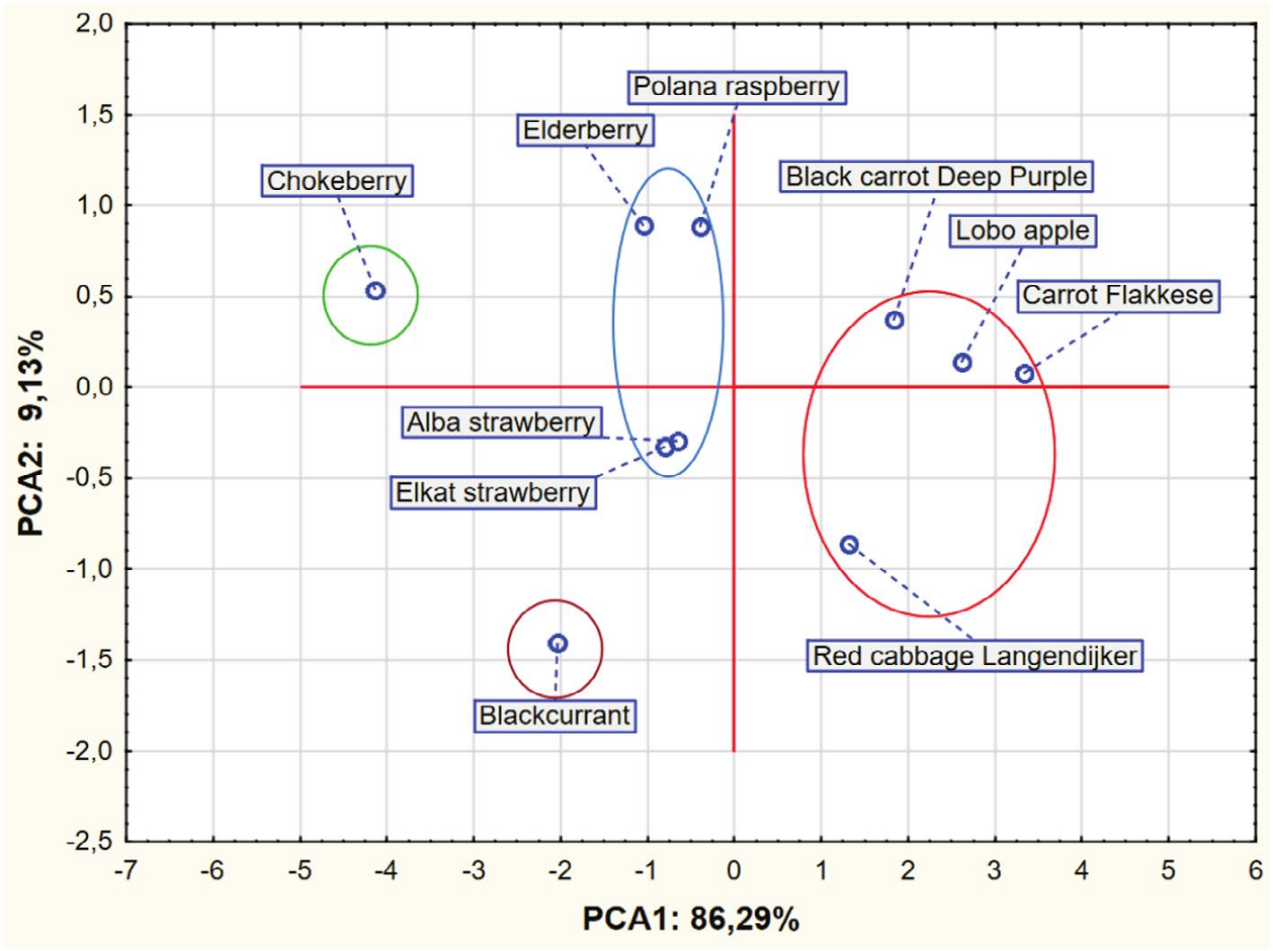
Chart of factor coordinates of cases (pomace) in the PCA model.

An analysis of Figure 1 indicates the existence of two main clusters of points representing the analyzed variants. The first group consists of the Black carrot “Deep Purple”, “Lobo apple”, “Carrot Flakkese” and “Red cabbage Langendijker” pomace, and the second group consists of the “Elderberry”, “Polana raspberry”, “Alba stawberry” and “Elkat strawberry” pomace. “Blackcurrant” and “Chokeberry” pomace created separate clusters.

**FIGURE 2:**
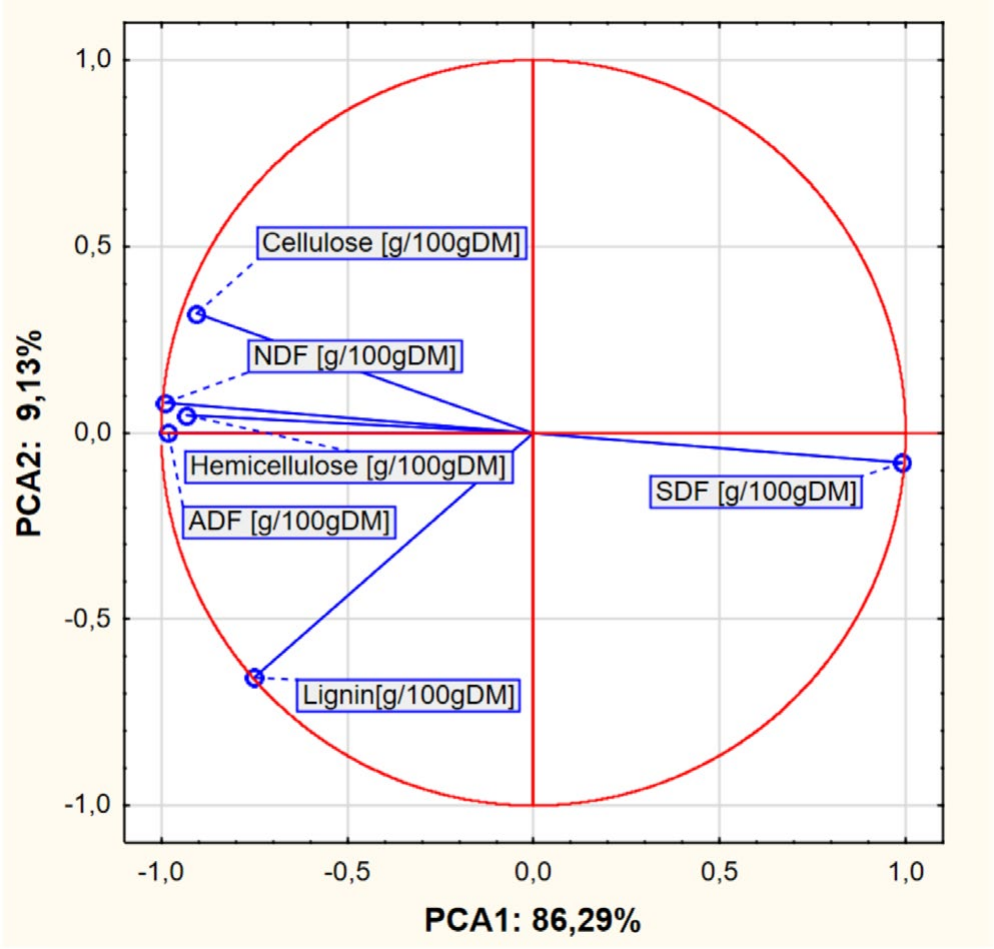
Chart of factor coordinates of variables in the PCA model.

When interpreting plots of factor coordinates of variables (Figure 2), attention should be paid to the length of the vectors and the angle between them. The longer the vector, the greater the share of a given feature (here PCA1 and PCA2). In turn, the smaller the angle between the vectors, the more similar the impact of these features (variables) on the analyzed components, i.e., the closer together the vectors (points) are located, the greater the positive correlation between the variables. The larger the angle the vectors form, the less correlated the variables are. If the vectors are perpendicular, then the variables are uncorrelated. If they form an angle close to 180°, they are negatively correlated.

A strong negative correlation was observed between SDF content and 3 variables: NDF, ADF, and hemicellulose content. A slightly weaker negative correlation was observed between SDF content and cellulose content. The weakest correlation was found between lignin content and all other analyzed variables.

## 4 Conclusions

In the context of environmental protection and food waste, pomace, as a waste product, can become a raw material for the production of functional food with increased DF content. As is well known, the composition of individual species and varieties significantly differs in the content of individual components. The studies conducted show that fruit pomace contained higher amounts of DF compared to vegetable pomace. Apple pomace was an exception, however. Most likely, such results can be explained by the use of the Lobo apple variety, which is less rich in DF. Chokeberry pomace contained the highest content of dietary fiber, while Flakkese carrot pomace showed the lowest DF values. Due to the significant amount of SDF fraction, apple pomace and two carrot varieties should be used for pectin recovery. Based on the PCA analysis, the similarity of individual raw materials in terms of the analyzed discriminants was found—mainly within two groups consisting of four raw materials. The features of two raw materials, i.e., blackcurrant and chokeberry, were significantly different from the others, which may be of practical importance when selecting specific raw materials for processing. The PCA analysis also showed mutual dependencies between the analyzed discriminants. This pomace can be successfully used to produce high‐fiber preparations, such as functional foods or dietary supplements.

## Author Contributions

**Agnieszka Nawirska‐Olszańska:** conceptualization (lead), data curation (lead), formal analysis (lead), methodology (lead), resources (lead), software (equal), validation (lead), visualization (lead), writing – original draft (lead), writing – review and editing (equal). **Maciej Oziembłowski:** conceptualization (supporting), formal analysis (supporting), software (equal), visualization (supporting), writing – review and editing (equal).

## Conflicts of Interest

The authors declare no conflicts of interest.

## Data Availability

The data that support the findings of this study are available on request from the corresponding author.
